# Author Correction: Hybrid CNN-LSTM model with efficient hyperparameter tuning for prediction of Parkinson’s disease

**DOI:** 10.1038/s41598-024-59648-6

**Published:** 2024-04-23

**Authors:** Umesh Kumar Lilhore, Surjeet Dalal, Neetu Faujdar, Martin Margala, Prasun Chakrabarti, Tulika Chakrabarti, Sarita Simaiya, Pawan Kumar, Pugazhenthan Thangaraju, Hemasri Velmurugan

**Affiliations:** 1 Department of Computer Science and Engineering, Chandigarh University, Chandigarh, Punjab India; 2 Amity School of Engineering and Technology, Amity University Haryana, Gurugram, India; 3 Department of Computer Engineering and Application, GLA University, Mathura, Uttar Pradesh India; 4 School of Computing and Informatics, University of Louisiana at Lafayette, Lafayette, USA; 5 Department of Computer Science and Engineering, Sir Padampat Singhania University, Udaipur, 313601 Rajasthan India; 6 Sir Padampat Singhania University, Udaipur, Rajasthan India; 7 Apex Institute of Technology, Department of Computer Science and Engineering, Chandigarh University, Mohali, Punjab India; 8 College of Computing Sciences & IT, Teerthanker Mahaveer University, Moradabad, Uttar Pradesh India; 9 Department of Pharmacology, All India Institute of Medical Sciences, Raipur, Chhattisgarh India

Correction to: *Scientific Reports* 10.1038/s41598-023-41314-y, published online 05 September 2023

The original version of this Article contained errors throughout the article, where several non-standard terms were used. These are now replaced with established scientific terminology. As a result, all instances of the terms “Parkinson’s illness”, “Parkinson’s infection”, and “Parkinson’s sickness” have been replaced with “Parkinson’s disease” throughout the text. Additionally, in the Literature Review section, the paragraph.

“Research proposed applying the Bidirectional long-transient memory method to catch time series dynamic elements of a discourse signal to distinguish Parkinson’s disease. The recommended way outflanks traditional AI models utilizing static highlights, as exhibited by tests using 10-overlay cross approval (CV) and dataset parting without difficulties from a similar individual covering.”

now reads:

“Research proposed applying the Bidirectional long-transient memory method to catch time series dynamic elements of a speech signal to distinguish Parkinson’s disease. The recommended way outflanks traditional machine learning models utilizing static highlights, as exhibited by tests using 10-overlay cross approval (CV) and dataset parting without difficulties from a similar individual covering.”

Secondly, the sentence,

“With an AUROC of 0.89 and a 10-overlay cross-approval precision of up to 82%, pair-wise positioning forecasts might be relied upon.”

now reads:

“With an AUROC of 0.89 and a 10-overlay cross-validation precision of up to 82%, pair-wise positioning forecasts might be relied upon.”

Thirdly, the sentence,

“For each casing of the films, act central assessment issues were extricated utilizing the profound learning system Open Pose, bringing about time-series signals for each main point.”

now reads:

“For each casing of the films, act central assessment issues were extricated utilizing the deep learning system Open Pose, bringing about time-series signals for each main point.”

Fourthly, the sentence,

“An irregular backwoods classifier was used to prepare an ordinal grouping framework (with one class for every conceivable rating on the UPDRS).”

now reads:

“Random forest classifier was used to prepare an ordinal grouping framework (with one class for every conceivable rating on the UPDRS).”

Fifthly, the paragraph,

“Six administered AI strategies were utilized for the order. The information was used to prepare the model, which was then tried utilizing approval measurements and a 10-overlay cross-approval procedure.”

now reads:

“Six supervised machine learning models were utilized for the order. The information was used to prepare the model, which was then tried utilizing approval measurements and a 10-overlay cross-validation procedure.”

Sixthly, the sentence,

“A deep multi-facet perceptron (DMLP) classifier was proposed by Research for use in research to monitor Parkinson’s disease progression using mobile devices.”

now reads:

“A deep multi-layer perceptron (DMLP) classifier was proposed by Research for use in research to monitor Parkinson’s disease progression using mobile devices.”

Seventhly, the sentence,

“Information expansion approaches were utilized to neutralize the spatial and fleeting predispositions in different development accounts, which considerably improved the presentation of the profound learning model.”

now reads:

“Information expansion approaches were utilized to neutralize the spatial and fleeting predispositions in different development accounts, which considerably improved the presentation of the deep learning model.”

And finally, the sentence,

“There were two arrangements of models, one built on a preparation and approval partner and the other surveyed in a free test companion by estimating the region under the bend (AUC) of the working trademark bends.”

now reads:

“There were two arrangements of models, one built on a preparation and approval partner and the other surveyed in a free test companion by estimating the area under the curve (AUC) of the working trademark bends.”

In addition, the code used for analysing the results has been implemented in IBM SPSS modeler, and not python as erroneously stated in the original version of the Article. Consequently, in the Material and Methods section, under the subheading ‘Extraction of Mel‑spectrograms’, the sentence,

“Figure 5 shows the Mel Spectrograms extraction results using the librosa python package on the PD dataset.”

now reads:

“Figure 5 shows the Mel Spectrograms extraction results using the SPSS software on the PD dataset.”

Under the subheading ‘Pseudo code for proposed CNN‑LSTM’, the sentence,

“The proposed CNN-LSTM model is implemented in the python language in Keras.”

now reads:

“The proposed CNN-LSTM model is implemented in the SPSS modeler software.”

In the Results and Discussion, the sentence,

“This research proposed a hybrid and existing ML model implemented using Python and various performance measuring parameters, i.e., accuracy, true positive rate, and false positive rate.”

now reads:

“This research proposed a hybrid and existing ML model implemented using SPSS modeler and various performance measuring parameters, i.e., accuracy, true positive rate, and false positive rate.”

And the sentence,

“In this work, the tenfold cross-validation is applied while executing these algorithms using Python.”

now reads:

“In this work, the tenfold cross-validation is applied while executing these algorithms using SPSS software.”

In the legend of Figure 5, the sentence,

“Mel Spectrograms extraction results using Libros python package on PD dataset.”

now reads:

“Mel Spectrograms extraction results on PD dataset.”

The original Article has been corrected.

